# Adoption of RTS, S malaria vaccine for children younger than 5 years in Rwanda: A budget impact analysis

**DOI:** 10.1016/j.rcsop.2021.100063

**Published:** 2021-08-16

**Authors:** Cyrille Ndikumukiza, Ismaeel Yunusa, Joseph Nkurunziza, Eric Chinaeke, Fahad Hezam Alshammari, Egide Abahuje, Saud Alsahali

**Affiliations:** a Analyda LLC, Boston, MA, United States of America; b College of Pharmacy, University of South Carolina, Columbia, SC, United States of America; c Ministry of Health, Saudi Arabia; d Simulation Education Fellow, STRATUS Center for Medical Simulation Brigham and Women's Hospital, Boston, MA, United States of America; e Department of Pharmacy Practice, Unaizah College of Pharmacy, Qassim University, Unaizah, Qassim, Saudi Arabia

**Keywords:** Rwanda, Malaria, Vaccine, RTS, S, Children, Budget impact

## Abstract

**Background:**

In Rwanda, malaria affects one in six children under five years old. Despite being preventable and treatable, malaria causes substantial morbidity, mortality, and economic burden on the Rwandan government and healthcare donors. Recently, the World Health Organization (WHO) agreed to consider the new malaria vaccine (RTS, S) as an additional prevention strategy. The Global Fund, a healthcare donor, is committed to donating more than fifty million US dollars over four years (2018–2021) to fight malaria in Rwanda. We estimated the potential budget impact of the adoption of RTS, S, into the Global Fund budget (as a case study) for malaria prevention in Rwanda.

**Methods:**

We developed a static budget impact model based on clinical, epidemiological, and cost (in US dollars) data from the literature, to assess the financial consequences of adding RTS, S to existing prevention strategies. Cost of treatment and prevention for the first year (without vaccine) was estimated and compared to the total cost after the fifth year (with vaccine). A one-way sensitivity analysis evaluated the robustness of the model.

**Results:**

For the 283,931children under 5 years at risk of malaria in Rwanda every year, the expected budget for first year (without vaccine) was $1,328,377.71 and for the fifth year (with vaccine) was $3,837,804, yielding a potential budget impact of $2,509,427. The cost of treating un-prevented malaria for the first year was $736,959 and for the fifth year was $61,413. The annual number of malaria treatments avoided increased from 10,095 children in the first year after introduction of vaccine to 36,701 children at the fifth year.

**Conclusion:**

With a potential budget impact of $2,509,427, the introduction of malaria vaccine for children under 5 years by Global Fund in Rwanda may be affordable when compared to the amount spent on treating children with malaria. Given that Malaria causes more harm than most parasitic diseases and disproportionally affects low-income populations, it is ethical to deploy all measures to control or eliminate Malaria, including vaccination.

## 1 Background

Malaria infection is a preventable and treatable disease transmitted by female Anophèles mosquitoes carrying malaria parasites.^1^ Almost half of the World's population is at risk of malaria, with pregnant women and children under five years old at most risk.^2^^,^^3^ It is estimated that 90% of all deaths due to malaria occur in sub-Saharan Africa and about 70% occur among children under five years.^4^ Malaria poses an enormous economic burden on Africa in addition to the loss of lives, with an estimated annual direct cost of US$12 billion and 1.3% GDP reduction due to disability and loss of labor hours.^1^ Available preventive measures to control infection are Long Lasting Insecticide Treated Nets (LLITN) or Insecticide Treated Nets (ITN), indoor residual spraying (IRS), and seasonal malaria chemoprevention (SMC),.^5^ Treatment options include Artemisinin-based combination therapy (ACT) as first-line treatment (adopted in most malaria-endemic parts of the African continent)^5^ and other anti-malaria medications.^2^

In randomized clinical trials of a new malaria vaccine (RTS,S), three doses of the vaccine prevented many clinical and severe malaria cases over 18 months.^6^, ^7^, 8. The pivotal trial enrolled 15,460 children in two age categories (6 to 12 weeks and 5 to 17 months of age) randomly assigned to either vaccination with either RTS,S or a non-malaria comparator vaccine.^9^ The trials' primary end point was vaccine efficacy against clinical malaria 12 months after vaccination in the first 6000 children who received all three doses of vaccine according to the trial protocol. In this trial, vaccine efficacy was evaluated after 250 children had an episode of severe malaria.^9^ The first result of the phase 3 clinical trial, suggested that the vaccine efficacy for 50.4% (95% confidence interval, 45.8 to 54.6) in the intention-to-treat analysis.^9^ Of note, to the best of our knowledge, there are currently no published real-world studies on the effectiveness RTS,S, and evidence from the clinical trial did not suggest the need for a booster dose. In 2015, the European Medicines Agency approved RTS, S for malaria prevention in children.^10^ In an analysis that considered the introduction and scale-up of the RTS, S malaria vaccine and the scale-up of LLITN, IRS and SMC, the RTS, S was found to be cost effective.^11^ Consequently, cost-effectiveness analysis determined that vaccination with RTS, S was more cost -effective in children compared to infants.^12^ Recently, the WHO agreed to consider the new malaria vaccine, RTS, S as an additional prevention strategy; however, the financial consequences of adopting this strategy and its affordability compared to existing approaches by governments and nongovernmental healthcare donors is unclear. The Global Fund, a healthcare donor, is committed to donating more than fifty million US dollars over four years (2018–2021) to fight malaria in Rwanda ^20^.

Rwanda, a rapidly-growing East African country, recorded a high coverage of LLITN use, which has contributed to a decline in malaria in the country.^13^ However, maintaining universal LLITN coverage is insufficient to protect citizens from disease completely.^13^ To mitigate this high malaria burden, more efforts and funding are needed to improve on existing prevention and treatment. Using Rwanda and the Global Fund budget as a case study, we aimed to develop a budget impact model (BIM) to estimate the consequence of the introduction of the RTS, S malaria vaccine into the Global Fund budget for malaria prevention in Rwanda.

## 2 Methods

### 2.1 Design

We developed a static BIM to assess the financial consequences after the introduction of RTS, S to existing prevention strategies in Rwanda, based on clinical,epidemiological, and cost data from literature.^14^

We constructed the BIM in Microsoft Excel with annual cycles, a base year before introduction of the vaccine, and four years following its introduction. A top-down approach was taken to estimate the cohort population size each year, with the population comprising prevalent cases of malaria in children five years and younger. We used a time horizon of 5 years for this BIM, which is informed by the usual short-term planning horizons by the budget holders and as recommended by the Belgian guidelines.^15^^,^^16^

**Fig. 1:**
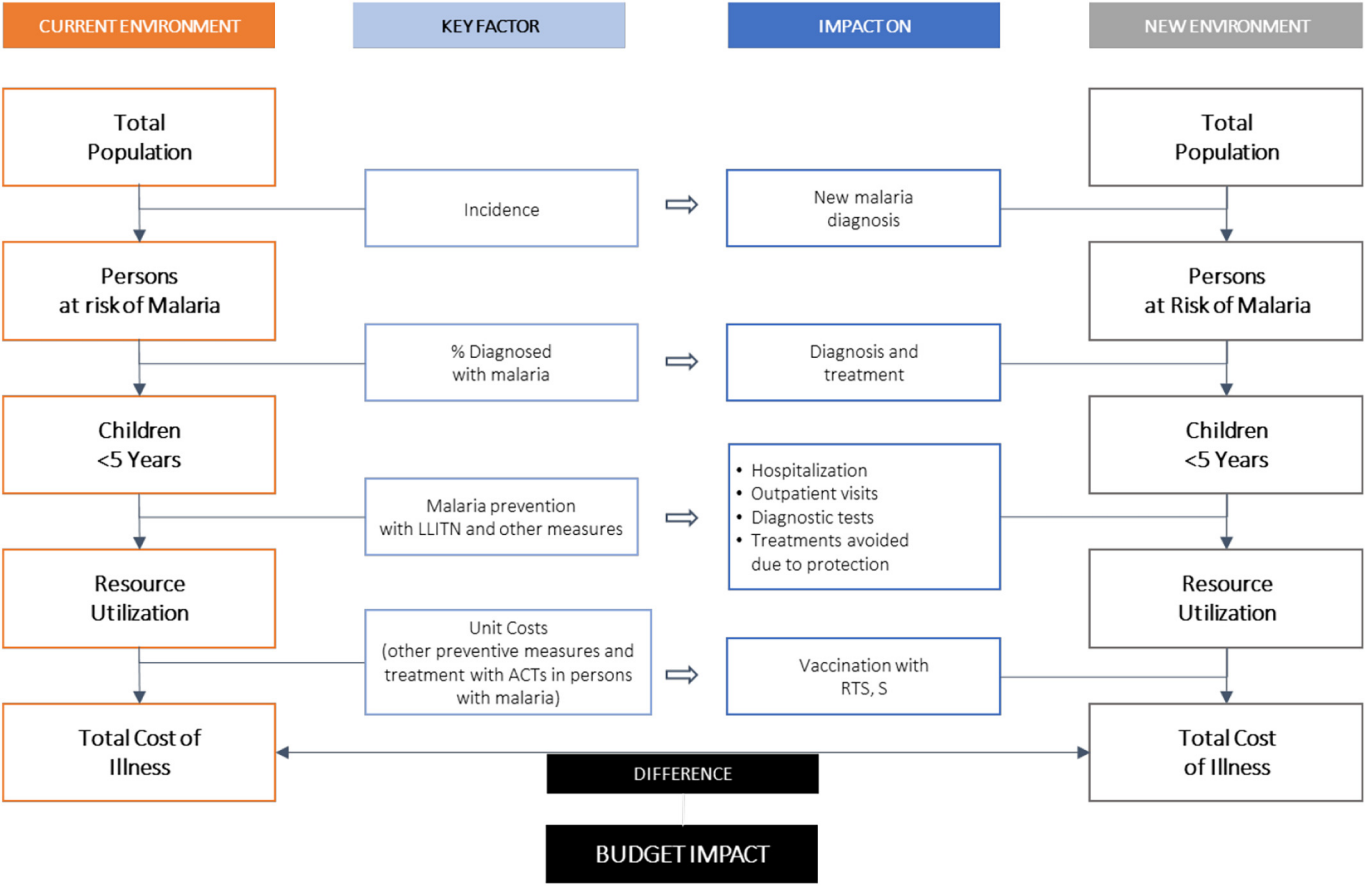
BIA framework. Adapted from: Sullivan SD, Mauskopf JA, Augustovski F, et al. Budget impact analysis-principles of good practice: report of the ISPOR 2012 Budget Impact Analysis Good Practice II Task Force. Value Health. 2014 Jan-Feb;17(1):5–14.

As illustrated in the BIM schematic (Fig. 1), we calculated the size of the population receiving treatment. For each intervention, we calculated total costs (i.e., LLITNs and cost of treatment ACT) and associated resource use on a per-person basis. Within each scenario (with or without RTS, S), we multiplied the per-person costs and resource use for each intervention by the population size. Once these values were calculated for each budget scenario, the differences between the budget scenario with RTS, S and the budget scenario without RTS, S represent the budget impact and resource use and health outcomes impact of RTS, S.

### 2.2 Inputs

According to Rwanda Malaria Indicator Survey (MIS) of 2018, the entire population of Rwanda is at risk of malaria and children under 5 years are prone to severe malaria due to lack of acquired immunity.^14^ This model included only children aged five and younger which is 2.0% of total population and corresponded to 210,319 (Table 2).^17^

We used the unit costs from the literature; the cost of prevention using LLITNs and cost of treatment ACT (Table 2). All costs were inflation-adjusted to 2018 US dollars using the Consumer Price Index from the US Bureau of Labor Statistics. We included ACT because it is the recommended first-line antimalarial drug and 99% of children under age 5 years received ACT according to 2017 Rwanda MIS report.^14^ The cost of vaccine was obtained from the literature.^11^ We first estimated the variation of percentage of patients on treatment before and after introduction of vaccine. The year zero ( base year [without vaccine]) was estimated and compared to the total cost after fifth year (with vaccine) (Table 1).

**Table 1:** Budget Impact on Cost of Malaria Prevention following the introduction of RTS, S. The cost of prevention increased from year 1 to 5, the treatment costs fell from year 1 to 5.

Estimation Cost	Current Year	Year 2	Year 3	Year 4	Year 5
LLITNs	$591,418	$665,346	$739,273	$813,200	$887,127
ACT⁎	$736,960	$552,720	$368,480	$184,240	$61,413
RTS, S	$0	$825,504	$1,651,008	$2,476,512	$2,889,264
Total	$1,328,378	$2,043,569	$2,758,760	$3,473,952	$3,837,804

Abbreviations: ACT, Artemisinin-based combination therapy; LLITNs, Long Lasting Insecticide-Treated Nets; RTS, S, New malaria vaccine.

⁎ Treatment of malaria for those who are not protected.

**Table 2:** Population and Unit cost values for interventions.

Variable	Value	Sources
**Population**		
Total population	10,515,975	Population and housing census (RPHC4) in 2012
% Incidence of malaria	2	Rwanda Demographic and Health Survey, 2016
**Unit Cost, US $**		
Long Lasting Insecticide-Treated Nets	7.03	White et al., 2011 and Winskill et al., 2017
Artemisinin-based combination therapy	5.84	White et al., 2011 and Winskill et al., 2017
Vaccine	39.25	White et al., 2011 and Winskill et al., 2017
Cost of treatment	23.31	Ettling and Shepard, 1991

A one-way sensitivity analysis was conducted to examine the robustness of the model findings. The following cost variables were included in the study and varied by ±20%: LLITN, ACT, and vaccine.

## 3 Results

### 3.1 Budget impact

**Fig. 2:**
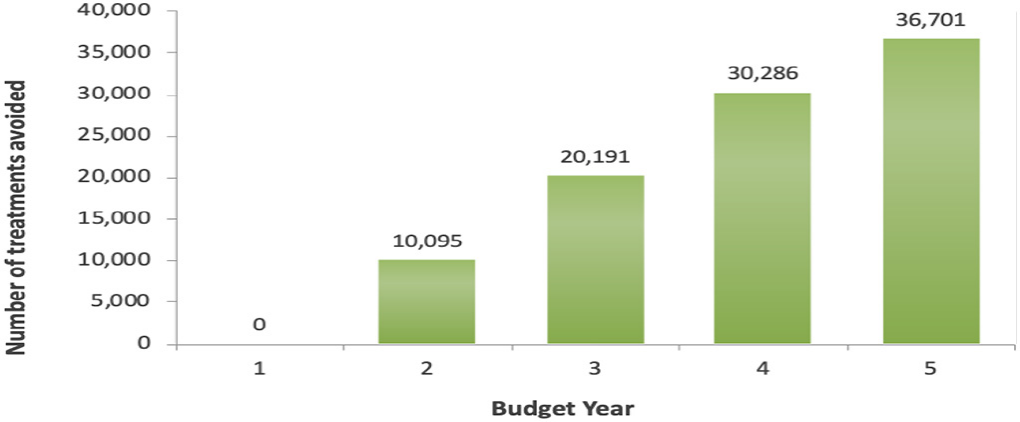
Number of treatments avoided following the RTS, S use in the period of 5 years. The total number of treatments avoided increased from 10,095 people in the second year after introducing vaccines to 30,286 people in the fifth year.

For the 283,931 children under 5 years at risk of malaria in Rwanda every year, the expected budget for first year (without vaccine) was $1,328,378 and for the fifth year (with vaccine) was $3,837,804, yielding a potential budget impact of $2,509,427. In the fifth year, the estimated total budget impact following the introduction of RTS, S was $3,837,804 (Table 1). The cost of prevention, including the vaccine, increased from year 1 to 5, while the cost of treating unprevented malaria with ACT substantially decreased (from $736,959 in the base year to $61,413 in the 5th year). Additionally, the total number of treatments avoided increased from 10,095 people in the year following the introduction of the RTS, S vaccine to 30,286 people in the fifth year (Fig. 2).

### 3.2 Sensitivity analysis

**Fig. 3:**
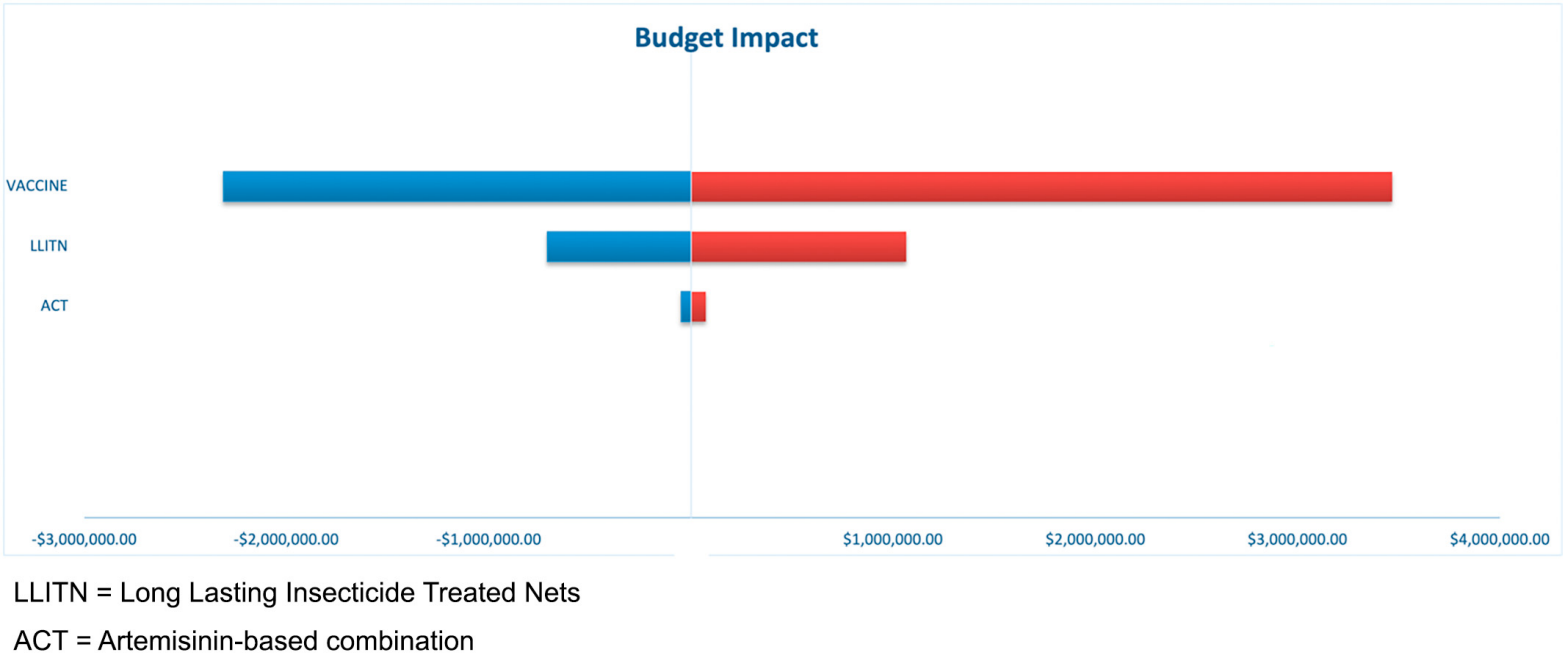
Tornado diagram for Sensitivity analysis. The input variables in the vertical axis were varied by ±20%: Vaccine, Lasting Insecticide Treated Nets, and Artemisinin-based combination. The horizontal axis presents the potential budget impact by varying the input variables.

In this budget impact analysis, the potential budget impact was influenced by the cost of the vaccine and LLITN. However, the cost of treating unprevented malaria with ACT had a minimal influence on the potential budget (Fig. 3).

## 4 Discussion

As the first vaccine approved for malaria, the introduction of RTS, S will require the health budget of Rwanda to be adjusted. African governments combine their efforts with international organizations to fight malaria. Among the largest donors in combating malaria are The Global Fund and United States' President's Malaria Initiative (PMI).^18^ These organizations are vital partners with the Rwanda government to eradicate malaria. The Global Fund, GAVI (Gavi, the Vaccine Alliance), and United funded the first phase of RTS, S malaria vaccine pilots held in select African countries.^19^ This study used the scenario where the Global Fund (the donor committed to donating more than fifty million US dollars in four years to fighting malaria in Rwanda.) is the sole sponsor of Malaria vaccination in Rwanda. ^20^

The estimated cost of vaccines in children under 5 was $2,889,264. We determined that, at the end of year 5, the cost of implementing a vaccine in Rwanda will increase the total spending by $2,509,427. After our calculation, the costs of implementing the vaccine program were higher compared to other interventions. The main goal of using the malaria vaccine is to avoid new malaria cases and reduce the direct and indirect cost of treatment. In this model 36,701 treatments were avoided after 5 years of vaccine use, corroborating the findings of a recent publication, where they found that 38,076 cases of malaria would be averted in Rwanda.^12^ The budget distribution will depend on numerous factors; however, by reducing the number of new cases of malaria seen in this model, we believe the introduction of the malaria vaccine will reduce spending in an extended period.

The model has some limitations. The cost of vaccination was assumed to be the same for infants and children; therefore, the additional cost for routine visits in the case of infants was considered minimal. The model also combines all types of malaria severity and assumes that the transmission was stable for the study period. This analysis did not consider the use of other types of interventions such as the IRS and SMC over the full five years of follow-up due to the limited information we had to input into the model. We did not find the cost associated with the burden of disease on families having sick children. This is a significant limitation and was due to a complex estimation of household income in Africa. The indirect cost of treatment can be worth tens of hundreds of millions of dollars based on culture and lifestyle. The lack of price transparency and cost estimation, in some cases, have made it impossible to run precise health economic analyses. In the future, additional studies will help to remove the confusion.

## 5 Conclusion

With a potential budget impact of $2,509,426, the introduction of malaria vaccine for children younger than 5 years by the Global Fund in Rwanda may be worthwhile in reducing the number of children diagnosed with malaria**.** Given that Malaria causes more harm than most parasitic diseases and disproportionally affects low-income populations, it is ethical to deploy all measures to control or eliminate Malaria, including vaccination.

## Funding

The authors received no financial support for the research, authorship and/or publication of this article.

## Declaration of Competing Interest

The author(s) declared no potential conflicts of interest with respect to the research, authorship, and/or publication of this article.
